# Long-Acting Growth Hormone Therapy in Pediatric Growth Hormone Deficiency: A Consensus Statement

**DOI:** 10.1210/clinem/dgae834

**Published:** 2024-12-03

**Authors:** Aristides Maniatis, Wayne Cutfield, Mehul Dattani, Cheri Deal, Paulo Ferrez Collett-Solberg, Reiko Horikawa, Mohamad Maghnie, Bradley S Miller, Michel Polak, Lars Sävendahl, Joachim Woelfle

**Affiliations:** Rocky Mountain Pediatric Endocrinology, Centennial, CO 80112, USA; Liggins Institute, University of Auckland, Auckland 1142, New Zealand; Genetics and Genomic Medicine Research and Teaching Department, UCL Great Ormond Street Institute of Child Health, London WC1N 1EH, UK; Centre de recherche CHU Ste-Justine, Université de Montréal, Montréal H3T 1C5, Canada; Disciplina de Endocrinologia, Departamento de Medicina Interna, Faculdade de Ciências Medicas, Universidade do Estado do Rio de Janeiro, Rio de Janeiro 20550-170, Brazil; Division of Endocrinology and Metabolism, National Center for Child Health and Development, Tokyo 157-8535, Japan; Department of Pediatrics, IRCCS Istituto Giannina Gaslini, 16147 Genova, Italy; Department of Neuroscience, Rehabilitation, Ophthalmology, Genetics, Maternal and Child Health University of Genova, 16147 Genova, Italy; Division of Endocrinology, Department of Pediatrics, University of Minnesota Medical School, M Health Fairview Masonic Children's Hospital, Minneapolis, MN 55454, USA; Service d’Endocrinologie, Gynécologie et Diabétologie Pédiatriques, Hôpital Universitaire Necker Enfants Malades, AP-HP, Université Paris Cité, 75015 Paris, France; Division of Pediatric Endocrinology, Department of Women's and Children's Health, Karolinska Institutet and Karolinska University Hospital, 171 77 Stockholm, Sweden; Department of Pediatrics and Adolescent Medicine, Friedrich-Alexander-University (FAU) Erlangen-Nürnberg, 91054 Erlangen, Germany

**Keywords:** children, consensus, growth hormone deficiency, long-acting growth hormone therapy, patient selection

## Abstract

**Context:**

Several long-acting growth hormone (LAGH) therapies have recently become available, but guidance on their usage in children with growth hormone (GH) deficiency is limited.

**Methods:**

International experts in pediatric endocrinology were invited to join a consensus group based on their expertise in treating children with daily GH and LAGH. The group comprised 11 experts from 10 countries across the world. Online group meetings were held in February to March 2024 followed by a 1-day in-person meeting in May 2024 to finalize the consensus recommendations. A targeted literature search approach was used to identify and share evidence ahead of the meetings. Formulations considered were limited to those with international populations in phase III pivotal trials and regulatory approvals in multiple countries.

**Evidence synthesis:**

Topics covered include patient selection and preference, dose adjustment, initiating and switching therapies, administration, adherence and missed doses, practical considerations, and knowledge gaps. LAGH formulations offer a potential advantage over daily GH injections for children with GH deficiency in terms of reduced injection frequency and treatment burden; this may also be associated with improved adherence and treatment outcomes over time. However, data on LAGH in pediatric GH deficiency are mostly limited to clinical trials, and long-term, real-world data are currently lacking.

**Conclusion:**

This article provides an international consensus on the use of LAGH therapy in children with GH deficiency to guide practitioners when considering these new treatment options for their patients. Long-term data are needed to fill current data gaps and allow the creation of comprehensive evidence-based recommendations.

Pediatric growth hormone (GH) deficiency was the first condition managed using daily injections of recombinant human GH (1-3). GH is also approved for children born small for gestational age (SGA) and patients with idiopathic short stature, Turner syndrome, Noonan syndrome, Prader–Willi syndrome, SHOX (short stature homeobox gene) deficiency, and chronic renal insufficiency (4). However, noncompliance with daily GH is common. A national study of GH adherence in New Zealand children reported an association with reduced linear growth (5). Two-thirds of patients missed more than 1 dose per week. Predictably, greater nonadherence was associated with a progressive fall in height velocity.

Several long-acting growth hormone (LAGH) therapies have recently become commercially available after pivotal phase III trials demonstrated noninferiority of these LAGHs to daily GH (6-9), but there is little guidance in the literature on their usage in the wider context of GH replacement in children. Given the efficacy and safety of daily GH injections, the question arises: Why use LAGH? This question is relevant both for children who are treatment naïve initiating GH therapy and for those who are treatment experienced with daily GH.

Long-acting therapy formulations are now in use in many therapeutic areas and have several potential advantages over short-acting formulations, including potentially improved adherence (10-12) and convenience, with a reduced therapeutic burden and a positive impact on quality of life (10, 13-15). Real-world data evaluating the impact of LAGH on adherence are currently lacking outside of the clinical trial environment.

Regarding treatment burden and quality of life, injectable therapies in children that require frequent administration have been associated with discomfort and impacts on daily life that extend beyond the timing of the injection itself (16-18). Examples of long-acting injectable pediatric endocrine therapies include depot GnRH analogs for precocious puberty (19), long-acting glucagon-like peptide-1 receptor agonists for type 2 diabetes and obesity (20), and somatostatin analogs for congenital hyperinsulinism (21). In terms of adherence, long-acting preparations have been shown to be more effective than daily formulations because of the improved adherence with long-acting formulations, particularly in patients at greater risk of nonadherence (22). When GH is considered, patients and/or parents/caregivers have shown a strong preference for LAGH preparations over daily GH formulations, mainly as a result of the reduced injection frequency (14, 23, 24). Perceived treatment burden is also lower with the long-acting preparations than with daily formulations (7, 14, 24), and caregivers believe adherence would be better with a LAGH preparation (23, 24).

The aim of this article is to provide an international consensus on the use of LAGH therapy in children with GH deficiency to guide practitioners when considering these new treatment options for their patients and to highlight the current gaps in knowledge for these formulations. It is likely that further experience with these preparations and real-world data will lead to further refinement of these guidelines in due course.

## Methods

An international group of experts in pediatric endocrinology was formed through invitation by the group's chairperson, Aristides Maniatis. Experts were invited based on their expertise in treating children with daily GH and LAGH and their involvement in publications and clinical trials of LAGH. The group comprised 11 experts from 10 countries across the world. Online group meetings were held in February to March 2024 to agree on the topics for inclusion followed by a 1-day in-person meeting in May 2024 for consensus building and statement development using a targeted literature search approach. All authors were involved in the development of these consensus statements, which were formulated using methodology based on a nominal group technique, whereby individuals responded to a series of questions. The responses obtained from all group members were then prioritized and agreed on. GH and LAGH formulations were limited to those with international patient populations in phase III pivotal trials and regulatory approvals in more than 1 country. Other LAGH formulations have been studied and approved in national populations and are not specifically discussed in this consensus because of their relatively limited availability.

### Search Strategy

Data sources included published data from pivotal phase III trials, conference abstracts, and the authors’ clinical experience because the peer-reviewed data on LAGH formulations are limited. Searches of PubMed were used to supplement the published data from pivotal phase III trials of LAGHs.

### Long-Acting Growth Hormone Formulations

The focus of this consensus is on 3 globally approved formulations of LAGH: lonapegsomatropin (Skytrofa), somapacitan (Sogroya), and somatrogon (NGENLA/Genryzon); listed in alphabetical order.

An overview of each product is shown in Table 1. Each formulation has a different mechanism to achieve extended duration (31). Lonapegsomatropin is a prodrug with transient PEGylation of GH. Somapacitan has a single-point mutation in the GH backbone (amino acid 101) with a noncovalent albumin-binding moiety attached. Somatrogon is a GH fusion protein with 3 carboxy terminal peptides of human chorionic gonadotropin. Somapacitan and somatrogon are supplied with a pen device, which permits adjustable dosing and multiple doses per pen, whereas lonapegsomatropin is used with an autoinjector and fixed-dose cartridges. Doses are weight based and administered once weekly for all 3 formulations. Dosing per kilogram varies by formulation, which is largely due to the varying molecular weight and pharmacokinetic–pharmacodynamic profile of the individual molecules. Appropriate selection of the pen device (somapacitan and somatrogon) for the patient's weight is important to minimize the need for multiple injections.

**Table 1.** Overview of long-acting growth hormone products available in more than 1 country

	LAGH		
	Lonapegsomatropin (25, 26)	Somapacitan (27, 28)	Somatrogon (29, 30)
Pivotal phase III trials	heiGHt (9): vs daily GH 0.034 mg/kg/day for 52 weeks N = 161 (2:1 randomization)	REAL 4 (7): vs daily GH 0.034 mg/kg/day for 52 weeks N = 200 (2:1 randomization)	NCT02968004 (8): vs daily GH 0.034 mg/kg/day for 52 weeks N = 224 (1:1 randomization) NCT03874013 (6): vs daily GH 0.025 mg/kg/day for 52 weeks N = 44 (1:1 randomization)
Clinical evidence*^a^*	Lonapegsomatropin demonstrated both noninferiority and superiority to daily GH for the primary endpoint AHV at 52 weeks using an ANCOVA and 2-sided 95% CI (*P* = .009) ANCOVA factors: treatment and sex ANCOVA covariates: baseline age, peak stimulated GH level, and height corrected for genetic potential (height SDS—average parental height SDS) (9)	Somapacitan demonstrated noninferiority to daily GH for the primary endpoint AHV at 52 weeks using an ANCOVA and pre-specified noninferiority margin of ≥−1.8 cm/year (NS) ANCOVA factors: treatment, sex, age group, peak GH level, geographic region, and sex by age group by region interaction term ANCOVA covariates: baseline height (7)	Somatrogon demonstrated noninferiority to daily GH for the primary endpoint AHV at 52 weeks using an ANCOVA and lower bound of the 2-sided 95% CI for the mean treatment difference (somatrogon—daily GH) of ≥−1.8 cm/year ANCOVA covariates: treatment, sex, age group, peak GH level, geographic region, and baseline height SDS (8). Somatrogon demonstrated noninferiority to daily GH for the primary endpoint AHV at 52 weeks using an ANCOVA and a point estimate of the mean treatment difference (somatrogon—daily GH) of ≥−1.8 cm/year ANCOVA factors: treatment and sex ANCOVA covariates: peak GH level, and baseline height SDS (6)
Extended duration mechanism of action	Transient PEGylation prodrug	Single-point mutation, hydrophilic spacer, noncovalent albumin binding	Fusion protein with 3 carboxy terminal peptides of hCG
Molecular weight of the active agent	22 kDa	23 kDa	40 kDa
Half life	25 hours	Approximately 34 hours	37.7 hours
Dosing	0.24 mg/kg/week	0.16 mg/kg/week	0.66 mg/kg/week
Manufacturer	Ascendis Pharma	Novo Nordisk Inc	Pfizer Ltd
Device	Autoinjector	Pen	Pen
Available dosage presentations	9 cartridges with weight-based brackets: 3 mg, 3.6 mg, 4.3 mg, 5.2 mg, 6.3 mg, 7.6 mg, 9.1 mg, 11 mg, and 13.3 mg	0.025 to 2 mg in a 5 mg pen 0.05 to 4 mg in a 10 mg pen 0.1 to 8 mg in a 15 mg pen	0.2 to 12 mg in a 24 mg pen 0.5 to 30 mg in a 60 mg pen
Maximum injection volume	0.605 mL	0.8 mL	0.6 mL
pH	5.0	6.8	6.6
Preservative	None	Phenol	Metacresol
Storage	Refrigeration; may be stored at room temperature (up to 86 °F; 30 °C) for up to 6 months	Refrigeration; may be stored at room temperature (up to 77 °F; 25 °C) for up to 72 hours	Refrigeration; may be held at room temperature (up to 90 °F; 32 °C) for up to 4 hours for a maximum of 5 times
Use window	4 hours after reconstitution	42 days after first use	28 days after first use
Body weight threshold requiring 2 injections	60.5 kg	50 kg	45 kg
Dosing window for isolated missed injections	±2 days	+3 days	+3 days

Abbreviations: ANCOVA, analysis of covariance; hCG, human chorionic gonadotropin; kDa, kilodalton; LAGH, long-acting growth hormone; NS, not significant; SDS, standard deviation score.

^*a*^From pivotal phase III trials.

The efficacy of each formulation in terms of height velocity at 1 year was noninferior to that of daily GH in prepubertal children with GH deficiency in the respective phase III trials for each formulation (6-9) (Table 1). However, given the relatively recent approvals for LAGH in pediatric GH deficiency (2021-2023), long-term and real-world data are currently lacking.

Two additional LAGH products, Eutropin Plus and Jintrolong, are approved in South Korea and China, respectively (32-38). These 2 products are not specifically discussed in this consensus statement because of their country-limited availability.

### Patient Selection and Preference

LAGHs are approved for the treatment of children with GH deficiency (starting from an age of 1-3 years according to country and product), and it is likely that most if not all of these children could benefit from a weekly administration regimen. Based on our clinical experience, we consider that the following groups may derive particular benefit from treatment with LAGH:

- Children at increased risk of poor adherence to daily GH, such as teenagers and those with previously documented poor adherence (39-41).
- Children taking multiple medications. Caution may be necessary in patients taking multiple medications given the potential for drug–drug interactions (25-30).
- Children with neurodiversity (including autism and attention-deficit/hyperactivity disorder) or other neurological or behavioral disorders who may have an increased tendency to be distressed by injections.
- Children with fear and anxiety associated with injections.
- Children with parents/caregivers who experience fear/anxiety associated with injections and the potential for “hurting” the child or affecting their relationship with the child.
- Children with more than 1 home because of the potential need for a supply of treatment in more than 1 location or to transport the treatment back and forth between residences.
- Children with frequent travel schedules outside of the home, such as sports competitions, extracurricular school activities (eg, musical groups), camping trips, and frequent sleepovers.
- Children whose families are socioeconomically disadvantaged and for whom GH treatment is an additional burden.

### Patients Who Were Not Included in the Published Clinical Trials for LAGH to Date

Although there is likely to be benefit in other growth disorders beyond GH deficiency, further trials of LAGH are needed in these disorders to demonstrate efficacy, safety, and appropriate dosing. Shared decision making between the treating endocrine practitioner, other health care practitioners involved in the patients’ care, and the patients and their caregivers is particularly important in these populations, which include the following:

- **Survivors of cancer and intracranial tumors associated with GH deficiency**. In survivors of cancer and intracranial tumors who may be considered for LAGH treatment, special consideration should be given to dosing and insulin-like growth factor (IGF)-1 monitoring. Recent evidence suggests that GH therapy can be initiated 6 months after last treatment for childhood craniopharyngiomas without increased risk of a new event (progression or recurrence) compared with a more delayed start to GH therapy (42). Although patients with pituitary tumors or craniopharyngioma remnants receiving daily GH do not need additional monitoring (43), future longer studies of LAGH are needed for these patients.
- **Prader–Willi syndrome.** Many patients with Prader–Willi syndrome will demonstrate GH deficiency and may be considered for LAGH treatment. These patients may have a predisposition to sleep apnea and metabolic abnormalities in addition to their short stature.
- **Very young patients**. LAGH therapies are indicated for children aged over 1-3 years (based on country and product). However, care should be taken in very young children with severe GH deficiency as the risk of hypoglycemia may be increased 1-2 days before each injection of LAGH (time of GH trough) (44). Clinical data are limited in very young patients as few patients aged under 3 years were included in the phase III trials of LAGH. Furthermore, these trials relied on reports of hypoglycemia symptoms and periodic fasting glucose concentrations but did not use continuous real-time glucose monitoring, so data on hypoglycemia are relatively lacking (7-9).
- **Pediatric non-GH deficiency states** such as SGA, idiopathic short stature, Turner syndrome, Noonan syndrome, SHOX deficiency, and chronic renal insufficiency. Phase II and III trials evaluating the use of LAGH in many of these conditions are ongoing, but results are still pending at the time of this publication. These specific conditions have traditionally required larger doses of daily GH, with higher associated IGF-1 levels, and have their own background genetic risk factors.

### Dose Adjustment

According to the respective product labels (25-30), starting dose recommendations are weight based for LAGH. However, freedom is given to adjust doses based on practitioner expertise and other clinical measures. Body surface area-based dosing recommendations are not currently available. As with daily GH therapy, dose changes for LAGH are at the discretion of the practitioner and may be based on IGF-1 levels, with potential adjustments for parameters such as an elevated body mass index, severity of GH deficiency, annualized height velocity, pubertal staging, estrogen supplementation, and bone age. To minimize the risk of transient hyperglycemia with LAGH (as reported in (45)), a lower initial starting dose may be considered based on ideal body weight rather than actual body weight for patients with obesity or who are otherwise already at risk for the development of hyperglycemia. A lower initial dose of LAGH may also be required for patients with increased risk of intracranial hypertension, severe GH deficiency, genetic or chromosomal abnormalities, or renal failure, as well as those receiving estrogen supplementation.

In the pivotal phase III clinical trials, dose was adjusted based on weight and reduced when IGF-1 levels were elevated or adverse events occurred. Dose reductions (15-20%) were performed in these trials based on elevated average IGF-1 levels greater than +2.0 standard deviation score (SDS) (7-9).

**Figure 1.**
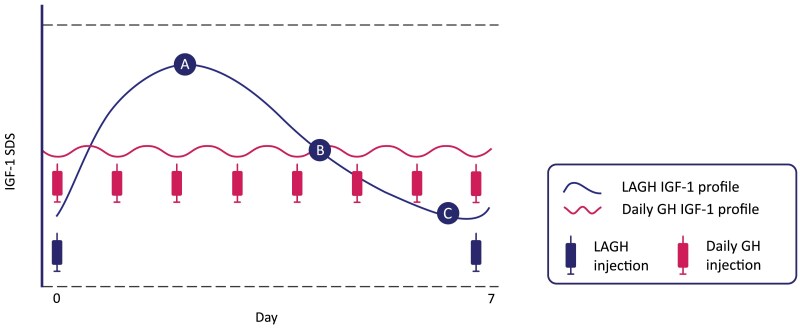
Conceptual representation of the pharmacodynamic differences between once-weekly long-acting growth hormone and daily growth hormone. A = peak, B = average, C = trough. Abbreviations: LAGH, long-acting GH; SDS, standard deviation score.

A conceptual representation of the pharmacodynamic differences (changes in IGF-1 levels) between LAGH and daily GH is shown in Fig. 1. In the European labels for LAGH, samples for IGF-1 are recommended to be drawn 4 days after the prior dose (27, 29) (4-5 days after dosing in the case of lonapegsomatropin (25)) and dose adjustments should be targeted to achieve average IGF-1 SDS levels in the normal range (between −2 and +2; preferably close to 0 SDS) (25, 27, 29). It is important to note both the day and the time of the laboratory sampling compared with the day and time of the last injection. If IGF-1 sampling is at day 4 (somapacitan and somatrogon) or day 4.5 (lonapegsomatropin), and levels are in the normal range, then no adjustment is needed to estimate the average IGF-1. If IGF-1 sampling occurs outside the specified 4- to 5-day window, the IGF-1 level can be adjusted using product-specific correction factors to estimate the average IGF-I level, as shown in Table 2. However, the larger the deviation from the average timing of IGF-1 sampling, the greater the uncertainty around the IGF-1 correction factor (46, 47). The estimated IGF-1 level from these calculations can be used to guide adjustments to the LAGH dose similar to the way that a random IGF-1 level is currently used to guide adjustments to the daily GH dose.

**Table 2.** IGF-1 correction factors IGF correction factors needed to estimate the average weekly IGF-1 SDS from samples taken at different time points during the week after the last injection of LAGH

LAGH	Time since last dose (days)														
	0	0.5	1	1.5	2	2.5	3	3.5	4	4.5	5	5.5	6	6.5	7
Lonapegsomatropin correction factor (46)	+1.22	+0.12	−0.39	−0.60	−0.67	−0.68	−0.62	−0.46	−0.21	No adjustment	+0.39	+0.64	+0.85	+1.07	+1.22
Somapacitan correction factor (47)	N/A	N/A	−0.80	−0.80	−1.0	−1.0	−0.5	−0.50	No adjustment	0	+0.70	+0.70	+1.1	+1.1	+1.1
Somatrogon correction factor (Pfizer Inc. New York, NY. 2024. Data on file.)	+1.8	+0.7	−0.1	−0.5	−0.7	−0.7	−0.6	−0.3	No adjustment	+0.4	+0.7	+1.1	+1.4	+1.6	+1.8

Abbreviations: IGF, insulin-like growth factor; LAGH, long-acting GH; N/A, not available; SDS, standard deviation score.

Prescribers should be aware that the per milligram calculation for weight-based dosing is different for each LAGH molecule and also differs from that of daily GH because of the unique pharmacokinetic/pharmacodynamic profile and molecular weight of each formulation. As such, direct milligram dose comparisons of the different molecules are not appropriate.

### Initiating and Switching Therapies

#### Initiating LAGHs in GH-naïve and GH-experienced patients

In our early clinical experience, patients already receiving daily GH may be less inclined to opt for LAGH than treatment-naïve patients because LAGH represents a new therapy compared with the traditional therapy to which they are accustomed. As more longer-term safety and efficacy data become available with LAGH products, we anticipate a continued shift towards preference for LAGH as late adopters become more comfortable with the new LAGH formulations.

#### Switching

The recommendation when switching between any 2 formulations of GH is to avoid overlapping dosing. As a general rule for children switching from daily GH, the first LAGH dose should be given the next day (or at least 8 hours) after their last daily GH dose. For patients already receiving LAGH switching to another LAGH formulation, the dose of the new LAGH should be given 7 days after the last dose of the prior LAGH.

### Administration

Regardless of which LAGH is used, more than 1 injection may be needed regularly for individuals weighing over 45 kg (somatrogon), 50 kg (somapacitan), or 60.5 kg (lonapegsomatropin), exceeding the maximum dose per application for the respective devices. This will happen for most children at some point during their treatment when their required dose cannot be supplied by a single pen injection or cartridge. Furthermore, to minimize end of pen medication wastage, more than 1 injection may occasionally be needed.

Autoinjector cartridges (lonapegsomatropin) have an advantage over pen devices (somapacitan and somatrogon) because the full dose is administered with each cartridge. Pen devices will more frequently require the administration of 2 doses per week based on partial doses remaining in the pen to avoid wastage. Pen devices (somapacitan and somatrogon) have an advantage over autoinjector cartridges (lonapegsomatropin) in their ability to have more dose graduations.

### Adherence and Missed Doses

There is a longer window (2-3 days) for a delayed dose of LAGH than with daily GH. However, it is important to ensure timely dosing of LAGH, since this can have implications for not only efficacy but also safety in terms of hypoglycemia risk. The general recommendation is to pick a specific day of the week as the injection day. If the injection cannot be given on that day, there is a ±2-day window (lonapegsomatropin) or a +3-day window (somapacitan and somatrogon) to give the dose as a “make-up” dose. The following week, injections should resume on the chosen specific day. The blood draw day (4-4.5 days after injection) should be taken into consideration when choosing the “dosing day”. It is imperative to not miss any doses of LAGH as it may have a large impact on efficacy.

### Practical Considerations

As LAGH products are relatively new, some practitioners and patients/caregivers may not be aware of LAGH products. Furthermore, not all formulations are available in every country. However, LAGH represents an important new treatment option that will become available to more children in the future.

Shared decision making between the practitioner, the patient, and their family is important when considering whether a LAGH will offer advantages to that individual over daily GH and, if so, which formulation and delivery device is right for them. The creation and use of tools and educational resources is recommended to empower patients and their caregivers to engage in shared decision making (48). When considering LAGH therapy, it is important for the practitioner to have a transparent and open discussion with the child and their family regarding the pros and cons of all potential therapies available to optimize acceptability and adherence (49).

Evaluation of nonadherence includes exploring the potential barriers and finding solutions with the child and their family/caregivers. If there is persistent nonadherence to LAGH, consideration should be given to third-party administration such as school or community nurses. LAGH can be given at any time of day, but time and injection site should be recorded, ensuring that injection sites are rotated to avoid lipoatrophy.

Depending on the country, access to LAGH may depend on registration, availability, price (to patient or health care service), or insurance/formulary.

### Specific Safety Considerations

The published safety profile of the LAGHs to date has been reassuring, with no new safety signals identified during follow-up periods of up to 5 years (7, 50-52). In the controlled phases of the pivotal phase III trials, LAGHs demonstrated comparable safety to daily GH, although 1 LAGH (somatrogon) was associated with more injection site reactions and pain than daily GH (7-9). In general, injection site reactions were mild or moderate with all LAGHs. The most commonly reported adverse events reported across clinical trials were headache, nasopharyngitis, pyrexia, gastroenteritis, respiratory tract infection, cough, and vomiting (7-9, 50-52). Antidrug antibodies to the LAGH formulations were observed in clinical trials, although these were not associated with any clinical effects (8, 53, 54).

### Knowledge Gaps

To date, published data on LAGH in children are largely limited to clinical trials in pediatric GH deficiency, and longer-term, real-world data are needed to answer outstanding questions on adherence, safety, and—importantly—efficacy, including body composition, cardiometabolic changes, and adult height outcomes. Furthermore, although dose adjustment is often based on the average IGF-1 SDS level, clinical implications of elevated peak and low trough IGF-1 levels are unknown. These need to be correlated with long-term clinical effect on neoplasia, cardiovascular outcomes, and glucose homeostasis.

None of the clinical trials included survivors of cancer and intracranial tumors associated with GH deficiency. This is an important population that requires real-world data. Data on the effect of LAGH on glucose control in very young children with severe GH deficiency using continuous glucose monitoring are also lacking. Careful consideration is needed before considering the use of LAGH in these potentially vulnerable groups.

Currently, the impact of LAGH on dose requirements for glucocorticoid and thyroxine are unknown, which represents a knowledge gap. Data on interaction of sex steroids (especially oral estrogens, which can reduce the biological effects of GH) and LAGH in adolescents are also needed. No data have been published on LAGH dosing requirements to optimize growth and bone health in adolescents with open epiphyses or on bone health and optimal dosing during the transition period from adolescence into adulthood. Only 1 LAGH (somapacitan) is currently approved for adult GH deficiency (defined as >18 years).

Future advances in autoinjector and pen technology may allow for objective recording of adherence and confirm improvements in adherence compared with daily GH.

Data are also lacking for pediatric non-GH deficiency states such as SGA, idiopathic short stature, Turner syndrome, Noonan syndrome, Prader–Willi syndrome, SHOX deficiency, and chronic renal insufficiency. Ongoing phase II and III trials are evaluating these conditions (excluding Prader–Willi syndrome and chronic renal insufficiency), but results are still pending at the time of this publication.

Patient registries are a key source of long-term safety and efficacy data, and several registries have been initiated. Long-term follow-up of patients into adulthood is necessary for monitoring of efficacy and safety. Data captured by registries may be limited in some cases, depending on the data source and whether they are specific to pediatric GH deficiency. The Global Registry For Novel Therapies In Rare Bone & Endocrine Conditions (GloBE-Reg) is a large, international registry open to those receiving any daily GH or LAGH formulation (55). The minimum data set for following children who are being treated with GH has been reported (56). The REAL10 study of somapacitan in children with GH deficiency is an active substudy within the GloBE-Reg registry (57), and the Pfizer Registry of Outcomes in Growth hormone RESearch (PROGRES) phase IV study of daily GH and LAGH in children (58) recently changed from an independent registry to be a substudy of GloBE-Reg. We recommend that individual national registries should establish processes for transfer of data to GloBE-Reg to maximize the quantity of data and range of patient types available in GloBE-Reg, which can be used to help to fill the data gaps outlined above.

Lonapegsomatropin has 2 independent registries for children with GH deficiency: SkyPASS in Europe and the United States, which began in 2023 (59), and SkybriGHt in the United States (60).

### Take-home Messages

1. The 3 globally approved formulations of LAGH for pediatric GH deficiency—lonapegsomatropin, somapacitan, and somatrogon—have demonstrated noninferiority to daily GH for efficacy in terms of annualized height velocity.
2. To date, the safety profile of these LAGHs is comparable to that of daily GH, with no new safety signals identified.
3. Given the unique pharmacokinetic/pharmacodynamic profile and molecular weight of each formulation, the per milligram calculation for weight-based dosing is different for each LAGH molecule and also differs from that of daily GH. Direct milligram dose comparisons of the different molecules are not appropriate.
4. For IGF-1 monitoring, it is important to note both the day and the time of the laboratory sampling compared with the day and time of the last injection. If IGF-1 sampling is at day 4 (somapacitan and somatrogon) and day 4.5 (lonapegsomatropin), this represents the average IGF-1, and no adjustment is needed. If IGF-1 sampling occurs outside the specified 4- to 5-day window, the IGF-1 level can be adjusted using product-specific correction factors.
5. Although there is likely to be benefit from LAGH in other growth disorders beyond GH deficiency, further trials are needed in these disorders to demonstrate efficacy, safety, and appropriate LAGH dosing. Shared decision making between the treating practitioner and the patients and their caregivers is particularly important in these populations.

## Conclusions

We provide consensus recommendations for the use of LAGH with respect to patient selection, dose adjustment, switching therapies, and practical considerations, based on the currently available data and clinical experience and expertise. The innovation of LAGH offers children and their parents/caregivers treatment options with reduced treatment burden, which may improve treatment adherence, quality of life, and clinical outcomes and address unmet needs. There may be particular benefits from LAGH for children or their caregivers who experience fear and anxiety associated with injections as well as children at increased risk of nonadherence due to other health issues, frequent travel schedules, split households, or socioeconomic disadvantages. Shared decision making between the clinician, caregivers, and patient is recommended when considering LAGH. Data on LAGH in pediatric GH deficiency are mostly limited to phase III clinical trials, and long-term data are therefore needed to fill current knowledge gaps and allow the creation of comprehensive evidence-based recommendations. Currently, there are also knowledge gaps for a number of patient groups who were not included in the phase III trials. Furthermore, long-term, real-world experience will provide ongoing safety and efficacy data, addressing the question of whether there is greater apparent efficacy (adult height) if fewer GH doses are missed with LAGH formulations. Patient registries will continue to be pivotal in generating these real-world data; increasing awareness of—and participation in—these registries is therefore paramount.

## Data Availability

Data sharing is not applicable to this article as no datasets were generated or analyzed during the current study.

## Abbreviations

GH: growth hormone
IGF: insulin-like growth factor
LAGH: long-acting growth hormone
SDS: standard deviation score
SGA: small for gestational age
