# Correction to: “Long-Acting Growth Hormone Therapy in Pediatric Growth Hormone Deficiency: A Consensus Statement”

**DOI:** 10.1210/clinem/dgaf125

**Published:** 2025-03-07

In the above-named article by Maniatis A, Cutfield W, Dattani M, Deal C, Collett-Solberg PF, Horikawa R, Maghnie M, Miller BS, Polak M, Sävendahl L, and Woelfle J (*J Clin Endo Metab*. 2024; doi: 10.1210/clinem/dgae834), there were errors in the Methods section and in Tables 1 and 2.

In the originally published article, in the Adherence and Missed Doses subsection of the Methods section, the fourth sentence read “If the injection cannot be given on that day, there is a ±2-day window (lonapegsomatropin) or a ±3-day window (somapacitan and somatrogon) to give the dose as a ‘make-up’ dose.” The “±” symbol before “3-day window” was incorrect, and it has been changed to “+” in the corrected version. In the corrected article, the sentence now reads “If the injection cannot be given on that day, there is a ±2-day window (lonapegsomatropin) or a +3-day window (somapacitan and somatrogon) to give the dose as a ‘make-up’ dose.”

In the originally published article, in Table 1, the two values under the “Somapacitan” and “Somatrogon” columns in the “Dosing window for isolated missed injections” row read “±3 days,” which was incorrect. In the corrected article, the information in both locations now reads “+3 days.”

In the originally published article, in Table 2, the data “−1.0” under the “3” column and the “Somapacitan correction factor” row was incorrect. In the corrected article, the value there now reads “−0.5.”

The article has been corrected online.

doi: 10.1210/clinem/dgae834

